# Diabetic Retinopathy: Two Faces of the Same Coin

**DOI:** 10.1100/tsw.2008.138

**Published:** 2008-11-22

**Authors:** Silvia Muñoz, Silvia Sanz, Núria Planas

**Affiliations:** Hospital Universitari de Bellvitge, Ophthalmology Department, L 'Hospitalet de Llobregat, Barcelona, Spain

**Keywords:** nonproliferative diabetic retinopathy, proliferative diabetic retinopathy, neovascular glaucoma, retinal hemorrhages, retinal new vessels

## Abstract

We report two contrasting cases of diabetic retinopathy to illustrate vascular and retinal abnormalities in this condition. In the first case, mild diabetic retinopathy is diagnosed in an asymptomatic patient with diabetes mellitus of recent onset. The second patient has severe diabetic retinopathy, causing vision loss as a consequence of poor metabolic control.

## CASE A

A 59-year-old woman was diagnosed with diabetes mellitus during an admission for urinary sepsis. She used lenses for high myopia. Best corrected visual acuity was 20/30 in the right eye (RE) and 20/40 in the left eye (LE). Anterior segment examination revealed bilateral nuclear cataracts. Fundus examination showed moderate nonproliferative diabetic retinopathy in both eyes (Figs. 1 and 2).

## CASE B

A 60-year-old woman with longstanding type-2 diabetes mellitus with poor metabolic control, hypertension, and mild renal insufficiency presented with pain and blurred vision in the RE. Fundus examination revealed multiple vascular and retinal abnormalities in the LE (Fig. 3). The lack of clear media in the RE did not allow proper examination, so eye ultrasonography was performed and showed signs of retinal traction. On the basis of these findings, severe proliferative diabetic retinopathy was diagnosed in the LE and neovascular glaucoma was diagnosed in the RE.

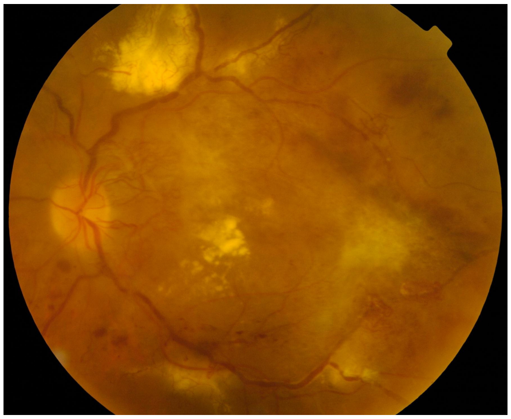

**FIGURE 3.** Case B. Retinal changes in the LE include extensive lipid exudate deposition in the macular area and superior temporal arcade, dot and blot intraretinal hemorrhages, and deep, shapeless subretinal hemorrhages. There is also marked venous beading and multiple new vessels around the optic nerve and both temporal arcades. Macular retina is thickened, suggesting the presence of macular edema.
